# Status epilepticus in POLG disease: a large multinational study

**DOI:** 10.1007/s00415-024-12463-5

**Published:** 2024-06-01

**Authors:** Omar Hikmat, Karin Naess, Martin Engvall, Claus Klingenberg, Magnhild Rasmussen, Eylert Brodtkorb, Elsebet Ostergaard, Irenaeus de Coo, Leticia Pias-Peleteiro, Pirjo Isohanni, Johanna Uusimaa, Kari Majamaa, Mikko Kärppä, Juan Dario Ortigoza-Escobar, Trine Tangeraas, Siren Berland, Emma Harrison, Heather Biggs, Rita Horvath, Niklas Darin, Shamima Rahman, Laurence A. Bindoff

**Affiliations:** 1 Department of Paediatrics and Adolescent Medicine, Haukeland University Hospital, Bergen, Norway; 2 Department of Clinical Medicine (K1), University of Bergen, Bergen, Norway; 3 European Reference Network for Hereditary Metabolic Disorders, Oslo, Norway; 4 Centre for Inherited Metabolic Diseases, Karolinska University Hospital, Stockholm, Sweden; 5 Department of Neuropediatrics, Astrid Lindgren Childrens Hospital, Karolinska University Hospital, Stockholm, Sweden; 6 Department of Molecular Medicine and Surgery, Karolinska Institutet, Stockholm, Sweden; 7 Department of Paediatric and Adolescent Medicine, University Hospital of North Norway, Tromso, Norway; 8 Paediatric Research Group, Department of Clinical Medicine, UiT, The Arctic University of Norway, Tromso, Norway; 9 Division of Paediatric and Adolescent Medicine, Department of Clinical Neurosciences for Children, Oslo University Hospital, Oslo, Norway; 10 Department of Neurology, Unit for Congenital and Hereditary Neuromuscular Disorders, Oslo University Hospital, Oslo, Norway; 11 Department of Neuromedicine and Movement Science, Norwegian University of Science and Technology, Trondheim, Norway; 12 Department of Neurology and Clinical Neurophysiology, St. Olav University Hospital, Trondheim, Norway; 13 Department of Clinical Genetics, Copenhagen University Hospital Rigshospitalet, Copenhagen, Denmark; 14 Department of Clinical Medicine, University of Copenhagen, Copenhagen, Denmark; 15 Faculty of Health, Medicine and Life Sciences, Department of Toxicology, University of Maastricht, Maastricht, The Netherlands; 16 Neurometabolic Disorders Unit, Department of Child Neurology/ Department of Genetics and Molecular Medicine, Sant Joan de Déu Children´S Hospital, Barcelona, Spain; 17 Department of Pediatric Neurology, Children’s Hospital and Pediatric Research Center, University of Helsinki and Helsinki University Hospital, Helsinki, Finland; 18 Stem Cells and Metabolism Research Program, Faculty of Medicine, University of Helsinki, Helsinki, Finland; 19 Research Unit of Clinical Medicine, University of Oulu, Oulu, Finland; 20 Department of Pediatric Neurology, Clinic for Children and Adolescents and Medical Research Center, Oulu University Hospital, Oulu, Finland; 21 Research Unit of Clinical Medicine, Neurology, and Medical Research Center Oulu, Oulu University Hospital and University of Oulu, Oulu, Finland; 22 Neurocenter, Oulu University Hospital, Oulu, Finland; 23 Movement Disorders Unit, Institut de Recerca Sant Joan de Déu, CIBERER-ISCIII, Barcelona, Spain; 24 European Reference Network for Rare Neurological Diseases (ERN-RND), Barcelona, Spain; 25 Norwegian National Unit for Newborn Screening, Division of Pediatric and Adolescent Medicine, Oslo University Hospital, Oslo, Norway; 26 Department of Medical Genetics, Haukeland University Hospital, Bergen, Norway; 27 Department of Clinical Neurosciences, University of Cambridge, Cambridge, UK; 28 Department of Pediatrics, Institute of Clinical Sciences, University of Gothenburg, Queen Silvia Children’s Hospital, Sahlgrenska University Hospital, Gothenburg, Sweden; 29 Mitochondrial Research Group, UCL Great Ormond Street Institute of Child Health, London, UK; 30 Metabolic Unit, Great Ormond Street Hospital for Children, NHS Foundation Trust, London, UK; 31 Department of Neurology, Haukeland University Hospital, 5021 Bergen, Norway; 32 European Reference Network for Hereditary Metabolic Disorders, London, UK; 33 European Reference Network for Hereditary Metabolic Disorders, Helsinki, Finland

**Keywords:** Epilepsy, Mitochondrial disease, *POLG*, Refractory status epilepticus

## Abstract

We aimed to provide a detailed phenotypic description of status epilepticus (SE) in a large cohort of patients with POLG disease and identify prognostic biomarkers to improve the management of this life-threatening condition. In a multinational, retrospective study with data on patients with POLG disease from seven European countries, we identified those who had SE. The age of SE onset, accompanying clinical, laboratory, imaging and genetic findings were analysed. One hundred and ninety-five patients with genetically confirmed POLG disease were recruited, of whom 67% (130/194) had epilepsy. SE was identified in 77% (97/126), with a median age of SE onset of 7 years. SE was the presenting symptom of the disease in 43% (40/93) of those with SE, while 57% (53/93) developed SE during the disease course. Convulsive SE was reported in 97% (91/94) followed by *epilepsia partialis continua* in 67% (56/84). Liver impairment 78% (74/95), ataxia 69% (60/87), stroke-like episodes 57% (50/88), were the major comorbidities. In the majority (66%; 57/86) with SE this became refractory or super-refractory. The presence of seizures was associated with significantly higher mortality compared to those without *(P* ≤ 0.001). The median time from SE debut to death was 5 months. SE is a major clinical feature of POLG disease in early and juvenile to adult-onset disease and can be the presenting feature or arise as part of a multisystem disease. It is associated with high morbidity and mortality, with the majority of patients with SE going on to develop refractory or super-refractory SE.

**Supplementary Information:**

The online version contains supplementary material available at 10.1007/s00415-024-12463-5.

## Introduction

Mitochondria are dynamic intracellular organelles with multiple vital functions, the most important being energy production. Oxidative phosphorylation (OXPHOS), the enzyme pathway responsible for ATP production, contains thirteen subunits encoded by mitochondrial DNA (mtDNA). In contrast, all the remaining mitochondrial subunits, together with more than a thousand other proteins required for mitochondrial structure and function, are encoded by the nuclear DNA [1, 2]. Pathogenic variants in any of the mtDNA or nDNA genes that encode proteins essential for mitochondrial structure and function may result in mitochondrial dysfunction and disease.

*POLG* is a nuclear gene that encodes the catalytic subunit of DNA polymerase γ, the enzyme that plays a vital role in mtDNA replication and repair [3, 4]. Pathogenic variants in *POLG* are one of the most common causes of inherited mitochondrial disorders [5], and are associated with a wide spectrum of clinical manifestations ranging from early-onset disease with therapy- resistant epilepsy and liver failure, to juvenile and adult-onset disease with epilepsy, stroke-like episodes (SLEs), ataxia, and peripheral neuropathy, and to late-onset disease with progressive external ophthalmoplegia (PEO) [5–8]. Patients with autosomal dominant disease present late in life with PEO, ptosis, and myopathy. Features such as ataxia, parkinsonism, premature ovarian failure, and cataract have also been described in this group of patient, but status epilepticus is generally not a feature [5].

Epilepsy is a common manifestation in patients with POLG disease, particularly in those with early and juvenile to adult-onset disease [8–11]. Focal seizures, commonly evolving into bilateral convulsive seizures, are the most common seizure semiology, with epileptiform discharges predominantly occurring over the occipital regions [8–10]. However, myoclonic seizures, epilepsia partialis continua and generalized SE are frequently reported [8, 10]. The presence of epilepsy is associated with higher mortality and increased morbidity, as the majority develop therapy-resistant epilepsy [8, 10, 12]. Patients may also present with refractory status epilepticus (SE) [5, 13, 14].

Despite the advances in the management of complex epilepsies and the availability of an increasing number of new anti-seizure medications (ASMs) with novel mechanisms of action, SE in patients with POLG disease remains a major therapeutic challenge. In pursuing effective treatment strategies, gaining a comprehensive understanding of the natural evolution of SE in patients with POLG disease and the response to available treatments is of paramount importance.

In this study, we aimed to describe the clinical course and systematically evaluate the current management of SE in a large number of patients with POLG disease. We also aimed to identify potential prognostic biomarker(s) which may predict the outcome in those with POLG disease and SE.

## Materials and methods

### Study design and population

In this international, multi-centre, retrospective study, patients with genetically confirmed POLG disease were recruited from 14 centres across seven European countries, including: Norway (Haukeland University Hospital, Oslo University Hospital, St. Olav’s Hospital and University Hospital of Northern Norway); United Kingdom (Great Ormond Street Hospital, London and Addenbrooke’s Hospital, Cambridge University Hospitals, Cambridge); Sweden (Centre for Inherited Metabolic Diseases, Karolinska University Hospital, Stockholm and Sahlgrenska Centre for Inherited Metabolic Diseases, Sahlgrenska University Hospital, Gothenburg), Denmark (Department of Clinical Genetics, Copenhagen University Hospital); Finland (Children’s Hospital, Helsinki University Hospital, and Clinic for Children and Adolescents, and Neurocenter, Oulu University Hospital); Netherlands (Department of Genetics and Cell Biology, Maastricht University, Maastricht) and Spain (Sant Joan de Déu Children’s Hospital, Barcelona).

Patients were stratified according to age of disease onset into three groups as previously described [8]: those with early-onset (< 12 years), juvenile to adult-onset (12–40 years), and late-onset disease (> 40 years). Patients with epilepsy were first identified and then grouped into those who developed SE and those who did not. Patients with SE were further subclassified according to the following three stages of SE [15–17]: established SE (SE that continues despite the first-line treatment with benzodiazepines), refractory SE (SE that persists despite at least two appropriately selected and dosed parental, first-line benzodiazepines and second-line treatment with antiseizure medications (ASMs)), and super-refractory SE (SE that continues at least 24 h after onset of third-line treatment, anaesthetics). Patients with early-stage SE (lasting 5–10 min) were not included due to incomplete data.

### Data collection

Patient data (demographics, age of disease, seizure and SE onset, clinical features including seizure and SE semiology and stage of SE, biochemical data at disease onset and later during the disease course, neurophysiological, neuroimaging and genetic findings, pharmacological and non-pharmacological treatment with ASMs and anaesthetics, ketogenic diet (KD) and immunomodulating therapies, response to treatment, and survival data) were systematically collected using a structured electronic case record form completed by the responsible investigator(s) in each participating centre and reviewed by the principal investigator, O.H. Whenever incomplete or unclear data were identified, further clarification was sought from the participating centre. Data collection for this study was completed December 2022.

Seizures, SE, and therapy-resistant epilepsy were defined using the International League Against Epilepsy (ILAE) definitions [16–19]. SLEs were defined as acute or subacute neurological dysfunction which might be preceded by prodromal symptoms such as migraine-like headaches, visual disturbances, and mental changes [20, 21]. Liver impairment was defined by the presence of two or more of the following in at least two different time points; elevated aspartate aminotransferase (ASAT)/alanine aminotransferase(ALAT), gamma-glutamyltransferase (GGT), bilirubin or ammonia, low serum albumin, or pathological histological findings on liver biopsy.

Data regarding SE management, including ASMs, anaesthetic agents, immunomodulators, and KD which had been used during different stages of SE, were collected. We looked specifically at the administration of valproic acid (VPA), as this is known to be associated with hepatopathy/ liver failure and contraindicated in patients with POLG disease [12]. The effects of immunomodulators and KD were also evaluated.

### Statistical analysis

Descriptive data analysis was performed using the statistical software package SPSS, version 23.0. Independent *t*-test was performed to compare between two groups. A two-sided P value less than 0.05 was statistically significant. For survival analysis, the endpoint was time to death, which was defined as the time in months from the date of seizure or SE onset to the date of death. Univariate survival analysis was performed using the log-rank test (Kaplan Meier method) to compare differences in survival time between categories.

## Results

### Demography

One hundred and ninety-five patients [males, *n* = 99 (51%)] with genetically confirmed POLG disease were recruited (Norway *n* = 92, Sweden *n* = 49, United Kingdom *n* = 23, Finland, *n* = 19, Denmark *n* = 8, The Netherlands *n* = 2, and Spain *n* = 2). The majority of patients were of Northern European origin (*n* = 184), while five patients were from the Middle East, two each from Cyprus and Spain, and one each from Croatia and Pakistan.

### Disease onset

Median age at disease onset (regardless of the presenting feature) for the whole study cohort was 16 years (range: birth to 71 years). Fifty percent of patients (*n* = 96) had disease onset before the age of 12 years (early-onset disease), 38% (*n* = 74) onset between age of 12- 40 years (juvenile to adult-onset disease), and 12% (*n* = 24) had late-onset disease (> 40 years). The definite age of disease onset was not available for one patient.

### Seizure and status epilepticus

Two-thirds of the patients recruited into this study, *n* = 130/194 (67%), developed seizures (no data regarding seizures in one patient), females *n* = 66/130 (51%) and males *n* = 64/130 (49%). Focal seizures and focal seizures evolving to bilateral tonic–clonic seizures were the most common seizure types (*n* = 114/194, 59%), followed by generalized tonic–clonic (*n* = 106/194, 55%) and myoclonic seizures (*n* = 84/194, 43%). Epileptic spasms were reported in one patient. Seizures were more frequently observed (*P* = 0.007) in patients with early onset disease, *n* = 79/130 (61%), as compared to those with juvenile to adult-onset disease,* n* = 49 (38%). Only two of those with late onset disease (1%) developed seizures.

**Fig. 1:**
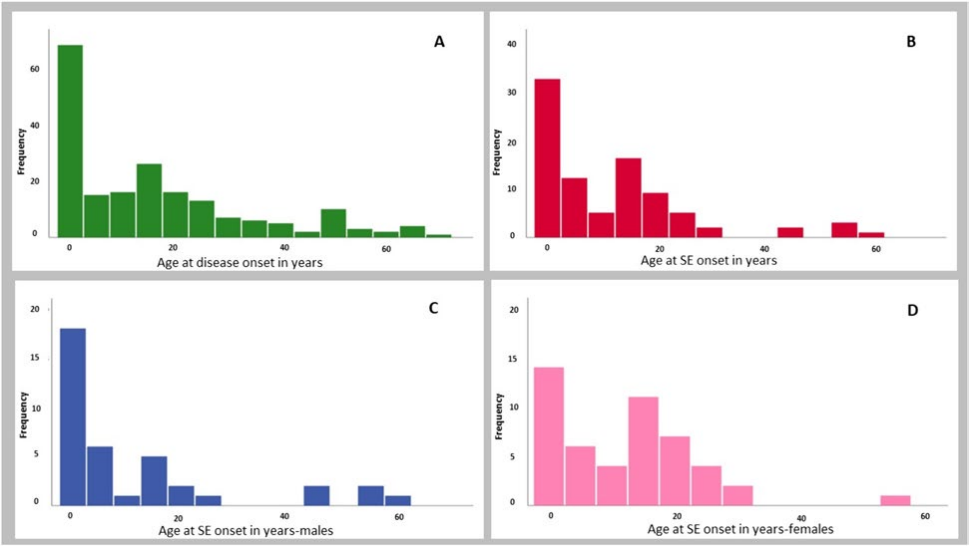
Age of disease onset and onset of status epilepticus for the whole study cohort of patients with POLG disease and also according to the gender. **A** Age of disease onset for the whole study cohort. **B** Age of onset of status epilepticus (SE). **C** Age of onset of SE in males. D: Age of onset of SE in Females

The majority (*n* = 97/126, 77%) of those with epilepsy developed SE at some point during the disease course (no data regarding SE in four patients with epilepsy), of those 57% (*n* = 55/97) were females and 43% (*n* = 42/97) males. Convulsive SE was reported in 97% (*n*=91/94) followed by *epilepsia partialis continua* in 67%(*n*=56/84). In the majority (66%,* n*=57/86) with SE this became refractory or super-refractory. The age of onset of SE showed a bimodal distribution with the first peak early in life (< 5 years of age) and the second peak around 14–16 years. This bimodal pattern was, however, gender dependent and was apparent only in females (Fig. 1). SE debut was apparently spontaneous in 68/97 (70%), following an infection episode in 27/97 (28%) and not clearly reported in 2/97 (2%). Median age of the first SE episode was 7 years (range: 4 months to 57 years). SE was more frequently observed (*P* =  < 0.001) in those with early onset disease.

**Fig. 2:**
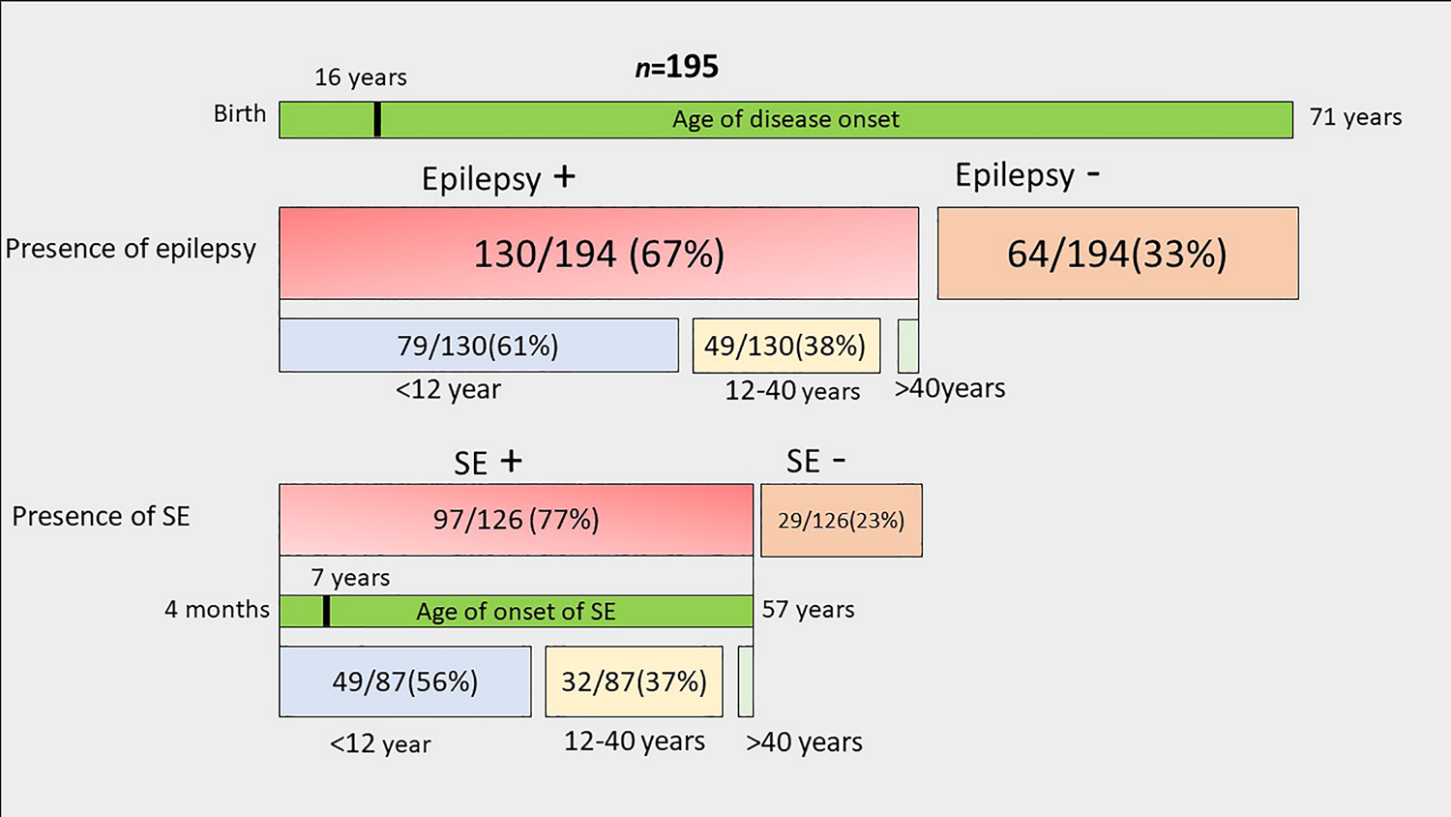
Summary of 195 patients with POLG disease stratified according to presence or absence of seizures, status epilepticus and age of onset of disease, seizures, and status epilepticus. Epilepsy developed in 67%. The majority of those with epilepsy were in the early (61%) and juvenile to adult-onset (38%) disease groups. Of those with epilepsy, 77% developed status epilepticus (SE) with a median age of SE onset of 7 years. SE is mainly manifested in early (56%) and juvenile to adult (37%) onset disease groups

SE was the presenting feature in 40/126 (32%) of those with epilepsy and in 40/93 (43%) of those with SE. Median time from disease onset to the first episode of SE in those who developed SE later during the disease course was 29 months (range 1 month to 47 years). Forty individuals had SE at disease onset; of these, 63% (*n* = 25/40) belong to the early onset disease group, and 37% (*n* = 15/40) to the juvenile to adult-onset group. Of the 53 individuals who developed SE later during the disease course, 60% (*n* = 32/53) belong to the early onset disease group, and 40% (*n* = 21/53) to the juvenile to adult group. None of those with late-onset disease presented with or developed SE; however, 6 patients with juvenile to adult-onset disease developed SE after the age of 40 years (Fig. 2).

### Major associated clinical features

In those with SE, liver involvement (*n* = 74/95, 78%), muscle weakness (*n* = 58/77,75%), ataxia (*n* = 60/87, 69%), and SLEs (*n* = 50/88, 57%) were among the most common clinical findings. A detailed description of the clinical findings is provided in Table 1.

**Table 1:** Major associated clinical features in patients with status epilepticus

Clinical features	No. (%)
A. Neurological features	
Muscle weakness	58/77 (75%)
Ataxia	60/87 (69%)
Stroke-like episodes	50/88 (57%)
Hypotonia	46/86 (53%)
Peripheral Neuropathy	38/77 (49%)
B. Gastrointestinal features	
Liver involvement	74/95 (78%)
Feeding difficulties	59/91 (65%)
C. Ophthalmological features	
Visual impairment	40/80 (50%)
Ptosis	18/91 (20%)
PEO	18/90 (20%)
D. Others	
Anaemia	67/88 (76%)

*PEO* Progressive external ophthalmoplegia. The number of the denominators reflects the available data for each specific variable

### Biochemical, electroencephalogram, and neuroimaging findings

Laboratory investigations in those with SE revealed increased lactate in blood in 41% (*n* = 24/58) and in cerebrospinal fluid (CSF) in 38% (*n* = 16/24) at disease onset, and later during the disease course in 68% (*n* = 47/69) and in 62% (*n* = 20/32), respectively. Aspartate and alanine aminotransferases were increased in 29% (*n* = 20/70) and in 23% (*n* = 18/78) at disease onset, in 81% (*n* = 67/83) and in 77% (*n* = 69/87) later during the disease course. One-third (*n* = 21/72, 29%) of patients with SE had a pathologically low haemoglobin value at disease onset. Anaemia developed in the majority of those with SE (*n* = 60/74, 81%) during the disease course. CSF protein or albumin was raised in more than two-thirds (*n* = 44/61, 72%) at disease onset and 83% (*n* = 30/36) later.

Electroencephalogram (EEG) showed epileptiform activity predominantly over the occipital lobes (*n* = 55/83, 66%), followed by the temporal lobes (*n* = 33/83, 40%), parietal lobes (*n* = 24/83, 29%), frontal lobes (*n* = 18/83, 22%) and multifocal (*n* = 18/83, 22%). EEG data were not available for 14/83 (17%) patients.

The available brain magnetic resonance images (MRI) were described as abnormal in 55/86 (64%) patients at SE onset. The most common (*n* = 55/77, 67%) MRI finding was cortical focal lesions (CFLs) manifesting as T2/FLAIR hyperintensities involving cortical and subcortical areas. Less frequently, T2-hyperintense thalamic lesions (*n* = 36/77, 47%) and generalized cerebral atrophy (*n* = 10/30, 33%) were observed. A description of MRI findings is provided in Supplementary Table 1.

### Genetic findings

Fifty-four percent (*n* = 52/97) of those who developed SE were compound heterozygous for pathogenic *POLG* variants, while 46% (*n* = 45/97) were homozygous. None of those with autosomal dominant disease developed seizures or SE.

The majority (*n* = 39/49, 80%) of those with early-onset disease were compound heterozygous, in contrast to those with juvenile to adult-onset disease, where the majority (*n* = 25/32, 78%) were homozygous. None of those who developed SE after the age of 40 years were compound heterozygous for *POLG* variants. Sixty percent (*n* = 24/40) of those who had SE at disease onset were compound heterozygous, while 53% (*n* = 28/53) of those who developed SE later during the disease course were homozygous (Supplementary Tables 2, 3). Further, of those who did not develop seizures, 30% (*n* = 19/64) were homozygous for pathogenic *POLG* variants, 61% (*n* = 39/64) were compound heterozygous, and 9% (*n* = 6/64) were heterozygous for *POLG* variants.

### Management of status epilepticus

The median number of ASMs used during a single SE episode was 6 (range 2–11); 62% (*n* = 53/85) of the patients were on more than six ASMs, 29% (*n* = 24/85) on four to six ASMs, and only 9% (*n* = 8/85) on fewer than three ASMs. ASMs, anaesthetic agents, immunomodulators, and KD which had been used during different stages of SE (established, refractory and super-refractory), stratified according to the age of disease onset and whether SE was the presenting feature of the disease or developed later during the disease course, are summarized in Table 2.

**Table 2:** Antiseizure medications and anaesthetic agents according to stage of status epilepticus

Status epilepticus Stage	SE management	Number of patients	Early onset	Juvenile-Adult onset	SE at onset	SE later
Established SE	Phenytoin, Fosphenytoin, Levetiracetam, Valproate, Phenobarbital, Midazolam	29/86 (34%)	18/29 (62%)	11/29 (38%)	9/33 (27%)	17/48 (35%)
Refractory SE	Midazolam, Thiopental, Propofol, clonazepam	19/86 (22%)	13/19 (68%)	6/19 (32%)	7/33 (21%)	11/48 (23%)
Super-refractory SE	Isoflurane gas, Prednisolone, ACTH, Immunoglobulin, Tacrolimus, Suxamethonium, KD	38/86 (44%)	22/38 (58%)	16/38 (42%)	17/33 (52%)	20/48 (42%)

*ACTH* adrenocorticotropic hormone; *KD* Ketogenic diet. The number of the denominators reflects the available data for each specific variable

Two-thirds (*n* = 130/195, 67%) of the whole study population developed some degree of liver impairment during the disease course, of whom only one-third (*n* = 46/130, 35%) had been exposed to VPA. Treatment with VPA was discontinued in 34 patients, reported to be effective in seizure control in two, and limited data were available for 10 patients. The reason for discontinuation of VPA was available for 21 patients; transiently raised liver enzymes in seven patients, liver failure in seven, no apparent effect on seizure control in six, and safety measure following the diagnosis of POLG disease in one patient.

KD was administered during an SE episode in 13 patients, with only two with juvenile to adult-onset disease reporting some effect on seizure control. Immunomodulators had been used in 24 patients (prednisolone in 16, immunoglobulin in four, ACTH (adrenocorticotropic hormone) in two, plasmapheresis and tacrolimus in one patient each); none of these were reported to achieve any positive clinical effect in seizure control or on survival. No difference (*P* = 0.19) in survival was observed in those who received immunomodulators compared to those who did not.

### Survival analysis

Of 97 patients with SE, 22/96 (23%) were alive at the time when data were collected, and one had been lost to follow-up. Patients with epilepsy had statistically significant (*P* =< 0.001) higher mortality compared to those without (Supplementary Fig. 1). However, there was no statistically significant difference in mortality in those with SE compared to those without in patients with epilepsy. Median time from SE onset to death was 5 months (range 42 days to 11 years) for those with early-onset disease, 6 months (range 14 days to 36 years) for those with juvenile to adult onset, and 2 months (range 27 days to 2 years) for those with juvenile to adult-onset who developed SE after the age of 40 years. Further analysis showed that the median time to death (Supplementary Table 4) in those with established SE was 6 months (range 34 days to 36 years), 5 months (range 42 days to 19 years) in those with refractory SE, and 4 months (range 14 days to 9 years) in those with super-refractory SE.

## Discussion

In this multi-centre study, we provide a detailed analysis of the impact of SE on patients with POLG disease using longitudinal data from the largest known international cohort of patients with this disease. The large number of patients provided a unique opportunity to improve our understanding of the clinical course of SE in POLG disease, evaluate the current management, enhance the statistical power of our study, and make it more likely to detect meaningful associations.

Our findings confirm that epilepsy is a major feature of POLG disease; more than two-thirds of the whole POLG study population developed epilepsy. This was particularly prevalent in those with early and juvenile to adult-onset disease. Importantly, we also found that the risk of developing seizures in those with late-onset disease was very low, with only two patients in this age group manifesting seizures, neither of whom developed SE.

SE was reported in more than three-quarters (77%) of patients with epilepsy and occurred in those with early and juvenile to adult-onset disease with a median age of onset of 7 years. None of those with late-onset disease developed SE, however, six patients (*n* = 6/74, 8%) with juvenile to adult-onset disease manifested SE after the age of 40 years. In a previous study [22], we showed that the onset of puberty has a negative impact on POLG disease in females; this is also true for SE, as the timing of SE onset showed a bimodal distribution in females with first peak early in childhood and the second around puberty (Fig. 1). SE was the presenting feature in one-third of those with epilepsy, and in patients with early-onset disease, it was the presenting feature in more than half of the patients. Further, in those with juvenile to adult-onset disease, SE was the presenting feature in more than one-third. These findings have important diagnostic implications for all patients presenting with SE: POLG disease is an important differential diagnosis in new onset SE in children and young adults.

We have previously suggested, based on data from a small series of patients, that prolonged seizures including SE, are closely associated with SLE [11] and can be linked to CFLs on MRI [20]. The results of this study confirmed our initial assumption as SLEs were one of the common accompanying clinical features and were reported in more than half of the patients with SE, and CFLs were the most common (67%) brain MRI findings in this group.

Our current study highlights the need for careful and regular monitoring of liver function tests, particularly during SE episodes, as liver impairment was one of the most frequent associated features in those with SE. The majority (78%) developed some degree of liver impairment, ranging from a transient increase in liver enzymes to fulminant liver failure. There is, currently, no evidence to support the possibility of liver dysfunction playing a role in epileptogenesis in POLG disease as patients can develop seizures and SE independently of clinically or biochemically detected liver dysfunction [8].

POLG disease is associated with blood–brain barrier dysfunction and raised CSF protein and/or albumin [23]. The presence of raised CSF lactate and CSF protein or albumin in patients with new-onset SE should raise the suspicion of POLG related SE in children and young adults, as we noticed these were elevated in 38% and 72% of these patients respectively at SE onset.

When we looked at the genotypes of those with SE, regardless of the age of onset, we found no significant difference between the percentage of those with compound heterozygous (52%) versus homozygous (48%) pathogenic *POLG* variants. However, we found a clear difference (*P* =< 0.001) when we stratified according to age of disease onset. The majority (80%) of those with early-onset disease and SE were compound heterozygous, while in those with juvenile to adult-onset disease, the majority (78%) were homozygous for pathogenic *POLG* gene variants. Interestingly, SE was the presenting feature in more than half of those with compound heterozygous variants, while in more than half of those with homozygous *POLG* variants, SE developed later during the disease course. We have shown in a previous study [8] that patients with compound heterozygous *POLG* gene variants have significantly worse survival compared to those with homozygous variants. Here, we showed again that patients with compound heterozygous for pathogenic *POLG* variants have a more severe disease course as compared to those with homozygous variants, as they often present earlier, frequently with SE as the presenting feature, and have worse survival. These findings are important not only for physicians responsible for the management of these patients, but also for the genetic counseling in POLG disease.

The results of this study clearly showed that treatment of SE in patients with POLG disease is challenging. Patients with early-onset disease had a higher risk of developing refractory and super-refractory SE compared to those with juvenile to late-onset disease. Further, in those with SE, refractory and super-refractory SE were the presenting feature of the disease in more than 70% of the patients. This finding highlights again that POLG disease should be considered in patients with new-onset refractory status epilepticus (NORSE).

More than two-thirds of those with SE proceeded to develop refractory or super-refractory SE, which was therapy-resistant, with patients receiving a mean of six ASMs. In addition to ASMs, most patients required multiple anaesthetic agents (a minimum of two agents) to achieve seizure control. The therapeutic effect of each individual anaesthetic agent was difficult to evaluate due to its combination with other ASMs and anaesthetic agents.

Administration of VPA was associated with liver impairment in different degrees, from a mild transient increase in liver enzymes to fatal fulminant liver failure. Although two patients in our study cohort reported a good response, VPA is absolutely contraindicated in patients with POLG disease [12].

KD may both improve mitochondrial function and has anti-inflammatory action [24]. A recent systematic review showed that KD may improve seizure control in patients with POLG disease [25]; however, we were unable to reach a firm conclusion about this due to the limited number (*n* = 13) of patients in our study who received KD, only two of whom reported some beneficial effect. Immunomodulatory therapies were used in 24 patients in our cohort without any obvious improvement in seizure control or survival. The effects of both KD and immunomodulatory therapies are difficult to evaluate as these had been used in a late, super-refractory stage of SE. Although not specific to mitochondrial related epilepsy, a systematic review of autoimmune encephalitis showed that delay in the administration of immunomodulatory therapies may be associated with a worse outcome [26]. The question of whether administration of immunomodulatory therapies and KD early during the course of SE will improve seizure/SE outcome is still unanswered and could best be resolved by a prospective randomized clinical trial.

Vagus nerve stimulation (VNS) is increasingly used in the treatment of refractory and super-refractory SE [27]. However, there are currently insufficient data confirming whether there is any benefit of VNS in patients with POLG disease. None of the patients in this study received VNS therapy during SE episodes.

Our study showed that the median time from SE onset to death was five months, and this correlated to the stage of SE, as those with refractory and super refractory SE had worse outcome and shorter elapsed time between SE onset and death. Measures targeting prompt and aggressive treatment of SE, including early administration of second- and third-line treatments, immunomodulatory therapies and KD, need to be investigated further. These improvement measures may reduce the risk of evolution into refractory and super-refractory SE and improve patient survival.

This study provides a detailed description of the clinical course of SE in patients with POLG disease and shows clearly that SE is a major feature of the disease in those with early and juvenile to adult-onset disease. The majority of those with SE developed refractory and super- refractory SE. Furthermore, the majority of patients with SE and compound heterozygous pathogenic *POLG* variants presented early (< 12 years of age), with SE as the presenting feature. The diagnosis of POLG disease should be considered in patients with NORSE.

### Supplementary Information

Below is the link to the electronic supplementary material.

Supplementary file1 (PDF 398 KB)

## Declarations

### Data availability

Anonymized data and the clinical record form for this study will be available upon reasonable request to the corresponding author.
