# Supplementary material for: Status epilepticus in POLG disease: a large multinational study
Source: J Neurol. 2024 Jun 1;271(8):5156–64. doi: 10.1007/s00415-024-12463-5 (PMC11319559; doi:10.1007/s00415-024-12463-5)
Supplement: Supplementary file 1 — Supplementary file1 (PDF 398 KB) [file 415_2024_12463_MOESM1_ESM.pdf]

Status epilepticus in POLG disease: a large multinational study
Journal of Neurology

Supplementary table 1: Brain MRI findings in patients with POLG disease and status epilepticus

Cerebral MRI findings	No. (%)
Cortical focal lesions	55/77 (67%)
Generalized cerebral atrophy	10/30 (33%)
Putamen lesions	1/73 (1%)
Caudate nucleus lesions	4/72 (5%)
Thalamus lesions	36/77 (47%)
Pons lesions	4/73 (5%)
White matter lesions	5/72 (7%)
Vermis lesions	4/72 (5%)
Dentate nucleus lesions	3/72 (4%)

Supplementary table 2: Number of patients with POLG and status epilepticus according to age of SE onset and genetic findings (compound heterozygous versus homozygous for *POLG* variants)

Age categories at SE onset	Compound heterozygous *POLG* variants	Homozygous *POLG* variants
< 12 years	39/49 (80%)	10/49 (20%)
12-40 years	7/32 (22%)	25/32 (78%)
> 40 years	0 (0%)	6/6 (100%)

Supplementary table 3: Number of patients with POLG disease and status epilepticus at disease onset or later according to genetic findings (compound heterozygous versus homozygous for *POLG* variants)

Onset of status epilepticus	Compound heterozygous POLG variants	Homozygous POLG variants
At disease onset	24/40 (60%)	16/40 (40%)
Later during disease course	25/53 (47%)	28/53 (53%)
Total	49	44

Supplementary table 4: Median time to death according to the stage of status epilepticus and medications which were administrated.

SE stages	Status epilepticus management categories	Median time to death (minimum/maximum)
Established SE	Phenytoin, Fosphenytoin, Levetiracetam, Valproate, Phenobarbital, Midazola	6 months (34days to 35 years)
Refractory SE	Midazolam, Thiopental, Propofol, clonazepam	5 months (42 days to19 months)
Super-refractory SE	Isoflurane gas, Prednisolone, ACTH, Immunoglobulin, Tacrolimus, Suxamethonium, KD	4 months (14 days to 9 months)

Supplementary Figure 1. Kaplan Meier curve comparing survival in those with and those without seizures.

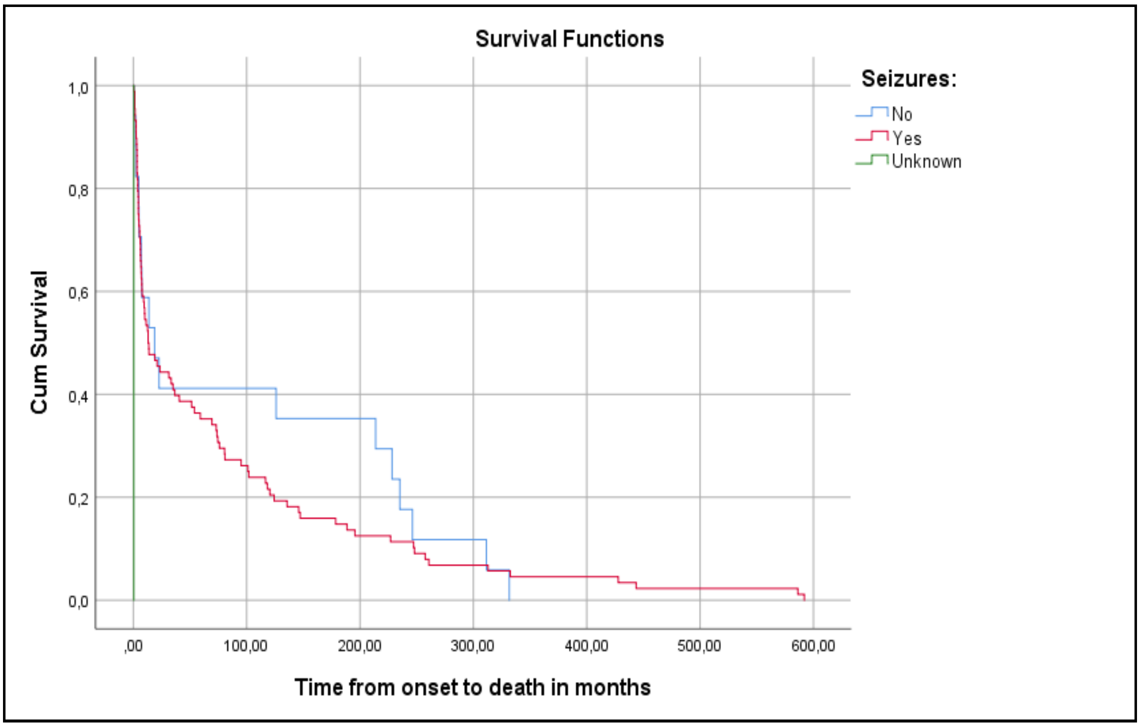

The presence of seizures was associated with a significantly worse survival (*P* =< 0.001)
